# Curcumin Prevents Epithelial-to Mesenchymal Transition-Mediated Ovarian Cancer Progression through NRF2/ETBR/ET-1 Axis and Preserves Mitochondria Biogenesis in Kidney after Cisplatin Administration

**DOI:** 10.34172/apb.2022.014

**Published:** 2020-09-19

**Authors:** Agian Jeffilano Barinda, Wawaimuli Arozal, Ni Made Dwi Sandhiutami, Melva Louisa, Nur Arfian, Normalina Sandora, Muhammad Yusuf

**Affiliations:** ^1^Department of Pharmacology and Therapeutics, Faculty of Medicine, Universitas Indonesia, Jakarta, Indonesia.; ^2^Metabolic, Cardiovascular and Aging Cluster, Indonesian Medical Education and Research Institute (IMERI), Universitas Indonesia, Jakarta, Indonesia.; ^3^Doctoral Program in Biomedical Sciences, Faculty of Medicine, Universitas Indonesia, Jakarta, Indonesia.; ^4^Faculty of Pharmacy, University of Pancasila, Jakarta, Indonesia.; ^5^Department of Anatomy,Faculty of Medicine, Public Health, and Nursing, Universitas Gadjah Mada, Yogyakarta, Indonesia.; ^6^Human Reproduction, Infertility, and Family Planning Cluster, Indonesian Medical Education and Research Institute (IMERI), Universitas Indonesia, Jakarta, Indonesia.; ^7^Dharmais Hospital National Cancer Center, Jakarta, Indonesia.

**Keywords:** Ovarian Cancer, Curcumin, Endothelin-1, ETBR, NRF2, Cisplatin-induced kidney injury

## Abstract

***Purpose:*** Ovarian carcinoma is one of the gynaecological malignancies that have the highest mortality rates due to its progressivity. Endothelin signalling plays a leading role in the progression of ovarian cancer through Epithelial-to-Mesenchymal Transition (EMT). Cisplatin commonly used as potent chemotherapy; however, its application hindered by its nephrotoxic effect. Curcumin, a turmeric-derived compound, has an anticancer property, as well as a renal protective effect. Moreover, curcumin augments the affinity of the antioxidant enzyme, while inhibits endothelin-1 (ET-1) signalling. The effects of curcumin on ovarian cancer progression and cisplatin-induced kidney injury remain unknown.

***Methods:*** Curcumin was used as a supplementary therapy together with cisplatin in Human Ovarian Cancer Cell line (SKOV3) and also in rodent-induced ovarian cancer. The kidney phenotype in the ovarian cancer rat model after cisplatin ± curcumin administration will also be analyzed

***Results:*** Co-treatment of cisplatin with curcumin enhanced the expression of a gene involved in apoptosis in association with NRF2 enhancement, thus activated ETBR-mediated ET-1 clearance in SKOV3 cell and ovarian cancer model in rat. Moreover, curcumin treatment improved mitochondria biogenesis markers such as PGC-1α and TFAM and prevented the elevated of ET-1-mediated renal fibrosis and apoptosis in kidney isolated from cisplatin-treated ovarian cancer rat.

***Conclusion:*** Curcumin could be potentially added as an anticancer adjuvant with protective effects in the kidney; thus, improves the efficacy and safety of cisplatin treatment in the clinical setting.

## Introduction

Ovarian cancer has the highest of fatality rates among all gynaecological malignancies, as two-third of the patients presented at the metastatic stage with more than 90% of the ovarian cancer cases derived from epithelial origin.^1-4^ Epithelial-to-mesenchymal transition (EMT) process defines as a bridging transformation of cancer into the metastatic phase. In contrast, the cells have lost their epithelial property with the loosening of cell junction with diminished E-cadherin and beta-catenin as epithelial markers. Therefore, the cell transforms into mesenchymal phenotypes with the enhancement of vimentin and N-cadherin markers.^5,6^

Endothelin-1 (ET-1) axis plays the most critical role in the epithelial plasticity in ovarian cancer. ET-1 is the very potent vasoconstrictor and mainly produced by the vascular endothelium. ET-1 has two types of receptors: Endothelin A (ETAR) and endothelin B (ETBR) receptor. Both of them are found in the vascular smooth muscle cell to regulate contractility, whereas ETBR is exclusively expressed in endothelial cell and acts as a receptor for ET-1 elimination.^7,8^

Strong evidence suggested a role of ET-1/ETAR in the development of ovarian malignancy. ET-1/ETAR highly expressed in many female malignancies such as cervical and ovarian cancer. ET-1/ETAR was overexpressed in the primary or metastatic ovarian cancer, as well as among ovarian cancer patient with ascites.^9-11^ Under physiological condition, ETBR plays as the counter-act for ET-1/ETAR mechanism by promoting ET-1 clearance, mediating apoptosis and inhibiting cell growth. However, it is still unknown whether ETBR could play some roles also in ovarian cancer cells^12^; nonetheless, the role of ETBR in the pathophysiology of ovarian cancer is remaining unidentified.

Nuclear factor erythroid-2-related factor 2 (NRF2) plays as the primary regulator of cytoprotective response to the endogenous and exogenous stresses; thus essential to prevent the cancer initiation and progression, either in healthy or malignant tissues.^13^ This pathway was showed to induce ETBR transcription and reduce ET-1 expression in endothelial cells isolated from human or rodent.^14^

Cisplatin (CIS) is a platinum-based chemotherapeutic drug that induces cytotoxicity through the kallikrein-kinin system and leads to inflammatory and oxidative stress.^15^ However, CIS has serious side effects such as hepatotoxicity,^16^ nephrotoxicity,^17^ ototoxicity^18^ and neurotoxicity.^18^ Therefore, studies to improve chemotherapy efficacy while reducing their side effects are rigorous to be able better to control the metastasis and progressivity of ovarian cancer.

Curcumin (CUR), a turmeric (*Curcuma longa*) compound with a yellow-coloured polyphenol, was reported as an anticancer agent through the disruption of cellular proliferation, survival, and apoptosis.^19-21^

CUR has believed to be a potent inhibitor for ET-1-mediated mitogenic and proliferative signalling in various cells including vascular smooth muscle, endothelial, and neural cells.^22-24^ CUR has been known as NRF2 activator and served as antioxidant properties suggesting the potential effect of CUR on ovarian cancer by activating antioxidant signalling.^25-28^ Our previous studies revealed that CUR prevented EMT through the suppression of oxidative stress in the breast cancer cell^29-31^ and prevented CIS-induced nephrotoxicity in the healthy rat by alleviating inflammation and eventually inhibiting apoptosis due to excessive oxidative stress.^32,33^ However, the role of CUR for preventing EMT process in ovarian malignancy and nephron-protector against CIS potentially through inhibiting ET-1 and oxidative stress has not been elucidated yet.

The CUR effects on ovarian cancer were investigated by observing the cancer progressivity and the transformation of the EMT process *in vitro* and *in vivo*. The capability of CUR to protect the kidney from CIS exposure in ovarian cancer model also studied through analyzing the expression levels of mitochondria biogenesis markers, and endothelin and its receptor markers.

## Materials and Methods

### Material

7,12-dimethylbenz(a)anthracene (DMBA) and CIS were obtained from Sigma (USA). CUR was obtained from Plamed Green Science Limited (China). CIS working solution was prepared in Phosphate Buffer Saline (PBS) for cell culture or in normal saline for in vivo study. CUR was suspended in DMSO for in vitro or carboxymethyl cellulose for in vivo analysis. All the solutions freshly prepared before the experiment. ETAR antibody was purchased from Santa Cruz, and EnVision+System+HRP anti-rabbit secondary antibody was purchased from Dako.

### Cell culture study

The SKOV3 ovarian cancer cell line was obtained from the American Type Culture Collection (ATCC, USA). The SKOV3 cells were cultured in RPMI medium (Gibco) supplemented with 10% Fetal Bovine Serum (FBS) (Gibco; Thermo Fisher Scientific), 1X Antibiotic-Antimycotic (100X) (ABAM) Solution (Gibco; Thermo Fisher Scientific) at 37°C in an incubator containing 5% CO2. For MTT assay, eight wells for each group were plated in a 96 wells dish and further treated with CIS or CUR in various concentrations as explained later in MTT viability assay. Another eight wells with PBS, DMSO, or PBS+DMSO in the medium used as the control for CIS, CUR, or CIS+CUR. For EdU Flow Cytometry analysis, a duplicate sample for each group was plated in a six wells plate and treated with CIS 3.75 µM ± CUR 5 µM. PBS+DMSO treated cells were used as control. For RNA extraction, a quadruplet sample for each group was plated in a 12 wells plate, induced with heat stress and followed with the same concentration of CIS ± CUR treatment as in EdU Flow Cytometry assay. Therefore, TRI Reagent and Direct-zol^TM^ RNA Miniprep Plus (ZYMO Research) used for RNA extraction and purification.

### Heat treatment-induced EMT transition in SKOV3 cells

The SKOV3 cells induced with insufficient heat treatment, as previously explained.^34^ After plating the cell in 12-wells plate, the cells were culture with the medium which previously prewarmed at 47°C, covered with parafilm, then put it in a water bath heated in 47 °C for 60 minutes. Afterwards, the cells were incubated with the fresh medium for next 24 hours as a recovery period. Therefore, the cells treated with CIS ± CUR for 48 hours and further proceed into RNA extraction.

### MTT viability assay

**Figure 1:**
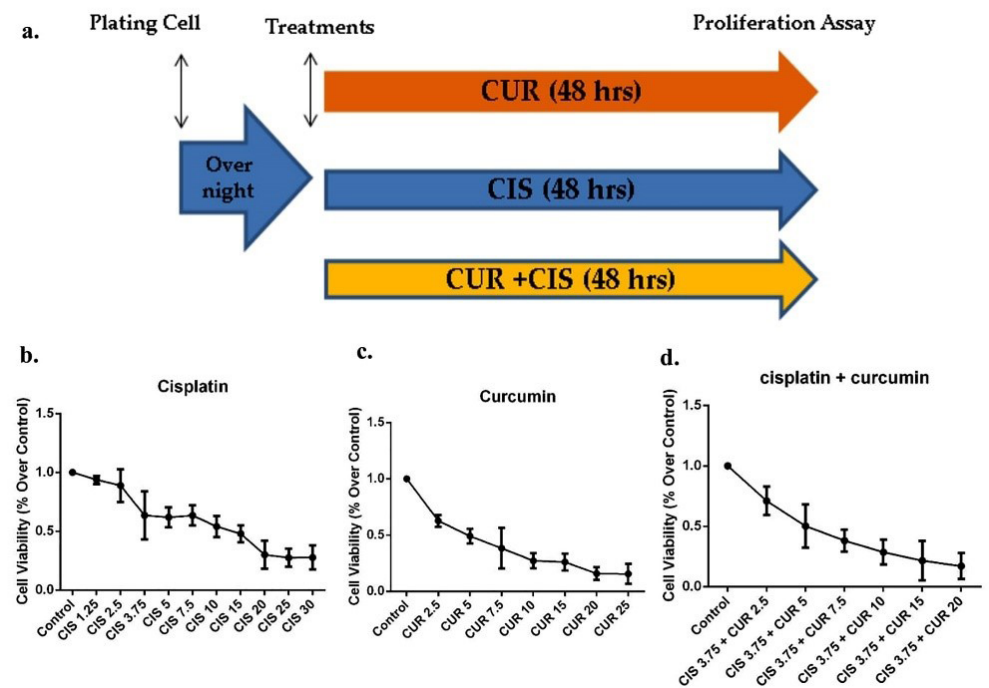
The MTT viability assay of cisplatin ± curcumin in SKOV3 ovarian cancer cell. (a) the timeline study of proliferation assay in SKOV3 cells treated with cisplatin ± curcumin (n=8 samples in each group). (b) Cell Viability in SKOV3 under various concentration of cisplatin (n=8 samples in each group). (c) Cell viability in SKOV3 under various concentration of curcumin. (d) Cell viability in SKOV3 under cisplatin at 3.75 μM and various concentration of curcumin (n=8 samples in each group). CUR: Curcumin; CIS: Cisplatin.

In brief, 5000 cells seeded in each well of 96 wells plate, and several concentrations of CIS (1.25-30 μM) or CUR (2.5-20 μM) were diluted and prepared in DMSO or PBS and treated into the cells until 48 hours for proliferation analysis (Figure 1a). The cellular growth capacity determined with Vybrant^®^ MTT Cell Proliferation Assay Kit (V-13154) (Thermofisher, USA). In brief, 10 μL of a reagent which contains 12 mM MTT stock solution was added in each well, and the cells were incubated at 37°C for 4 hours, and afterwards, additional of 100 μL of the SDS-HCl solution was added and proceeded into final incubation for overnight at 37°C. The proliferation data were analyzed by measuring colour intensity at 570 nm and calculated by subtracting with colour intensity of PBS, DMSO, or PBS+DMSO as control for –CUR, –CIS, and –CIS+CUR in medium, respectively.

### EdU flow cytometry assay

The flow cytometry procedure was performed according to the manufacturer’s protocol. The SKOV3 cells were suspended with a fresh medium and labelled with EdU by adding 10 µM of Click-iT^®^EdU for 1-2 hours. After cells washing with 3ml of 1% Bovine Serum Albumin (BSA) in PBS, the SKOV3 cells were centrifuged and resuspended the cells with 1% BSA in PBS and further permeabilized with 100 µL of 1X Click- iT^®^saponin for 15 mins. Wash the cells with 1% BSA in PBS, added 0.5 mL of Click-iT^®^EdU reaction cocktail, and analyzed the cell using flow cytometer.

### Animal study

Five weeks old female Wistar rats obtained from Institute of Research and Development, Ministry of Health of Republic Indonesia and were acclimatized for the initial one week in an animal chamber with a temperature of 25°C, humidity: 65%, and 12-hours light cycle per day from 07.00 until 19.00. Rats were housed in the animal chamber (1 rate in each cage) and fed a standard pellet with ad libitum access in food and water. The generation of ovarian cancer rat model was adopted from our established model as previously described.^35^ The Cancer model was performed by directly implanting the silk-coated with DMBA into the ovarian organs under surgery with intraperitoneally mixture injection of ketamine hydrochloride (75 mg/kg BW) and xylazine (8.8 mg/kg BW). Moreover, kept the rats for 20 weeks and followed with the treatment of CIS (4 mg/kg BW) once in a week intraperitoneally ± CUR (100 mg/kg BW) orally everyday for additional four weeks. Therefore, the samples divided into four groups (n=6, each) which consist of Sham/control group, ovarian cancer + vehicle group, ovarian cancer + CIS treatment group, and ovarian cancer + CIS + CUR treatment group. Afterwards, the rats were terminated, and the ovarium coated with DMBA-silk or kidney were collected and used for –RNA extraction and -histological analysis, respectively. Also, Blood was collected and incubated at room temperature for 60 minutes, then proceed with centrifuged at 1500 × *g* for 10 minutes and collected the blood serum for plasma urea and creatinine analysis.

### Quantitative PCR

RNAs derived from SKOV3 ovarian cancer cells or ovarium were purified using Direct-zol^TM^ RNA Miniprep Plus (ZYMO Research) according to the manufacturer’s protocol. cDNA from the samples was synthesized from ~0.5 μg of total RNA using reverTra Ace qPCR RT Mastermix with gDNA remover (TOYOBO). Therefore, the PCR reactions prepared using Thunderbird^®^ Sybr qPCR mix (TOYOBO) followed by the real-time PCR analysis using LightCycler^®^ 480 Instrument II (Roche Applied Science). Quantification of genes expression were using the delta-delta CT (∆∆CT) method, which normalized with Beta Actin mRNA. The Human and Rat Primers used shown in Table S1 and S2 (see Supplementary file 1).

### Histology analysis

Ovarium with DMBA-coated silk and kidney was isolated and fixed with 10% formalin buffer for 24 hours, followed with paraffin embedding. 4 µm sections of ovarium used for ETAR Immunohistochemistry; whereas 5 µm sections of kidney used for PAS and Masson’s Trichome Staining.

### Immunohistochemistry (IHC)

ETAR IHC performed as previously described. After deparaffinization, 4 µm paraffin sections of ovarium were incubated with 3% H_2_O_2_ in PBS for 5 minutes to reduce the endogenous peroxidase. Therefore, sections were incubated with anti-ETAR antibody (1:100) (SC33535, Santa Cruz) at 4°C for overnight and followed with EnVision+System+HRP anti-rabbit secondary antibody (K4002, Dako) (1:200) for 60 minutes at room temperature. Finally, immune-positive cells were visualized by incubating with 3,3’-diaminobenzidine. Hematoxylin staining used for counterstaining the sections.

### Statistical analysis

The Data are presented as mean ± SD. Disparities of groups were investigated by using two-tailed student’s t test and differences among more than three groups were analyzed by analysis of variance (ANOVA) followed by Tukey or Bonferroni methods for post hoc analysis. A value of *P* <0.05 was considered statistically significant. GraphPad Prism version 6.01 (GraphPad Software, San Diego, CA, USA) was used for statistical analysis.

## Result and Discussion

### Viability assay of CIS ± CUR in SKOV3 cell

We initially identified whether CUR reduces cell viability and induces apoptosis in ovarian cancer cells using SKOV3 cells. We established an outline strategy of SKOV3 cells treated with a single treatment of CUR or CIS and a combination of both treatments (Figure 1a). As shown in Figures 1b, c, proliferation cell capacity was gradually decreased at CUR or CIS treatment alone in a dose-dependent manner. Similarly, other groups and we have previously demonstrated that CUR suppresses cell growth in cancer model both in vitro and in vivo.^20,21,29,30^ Moreover, we further confirmed that CIS at 3.75 µM and CUR 5 µM (CIS+CUR) significantly induced the half-maximal inhibitory concentration (IC50) in SKOV3 cell. (Figure 1d). Therefore, we used this compound formulation for subsequent experiments. Our data showed that CUR treatment alone was likely to be more potent to induce apoptosis in SKOV3 cells compared with those in CIS alone. Other studies revealed the possibility of CIS resistance in the ovarian cancer cell.^36,37^ However, future works are needed to analyze that resistance using specific CIS resistance of SKOV3 cell.

### CUR induces apoptosis activity in SKOV3 Cell

**Figure 2:**
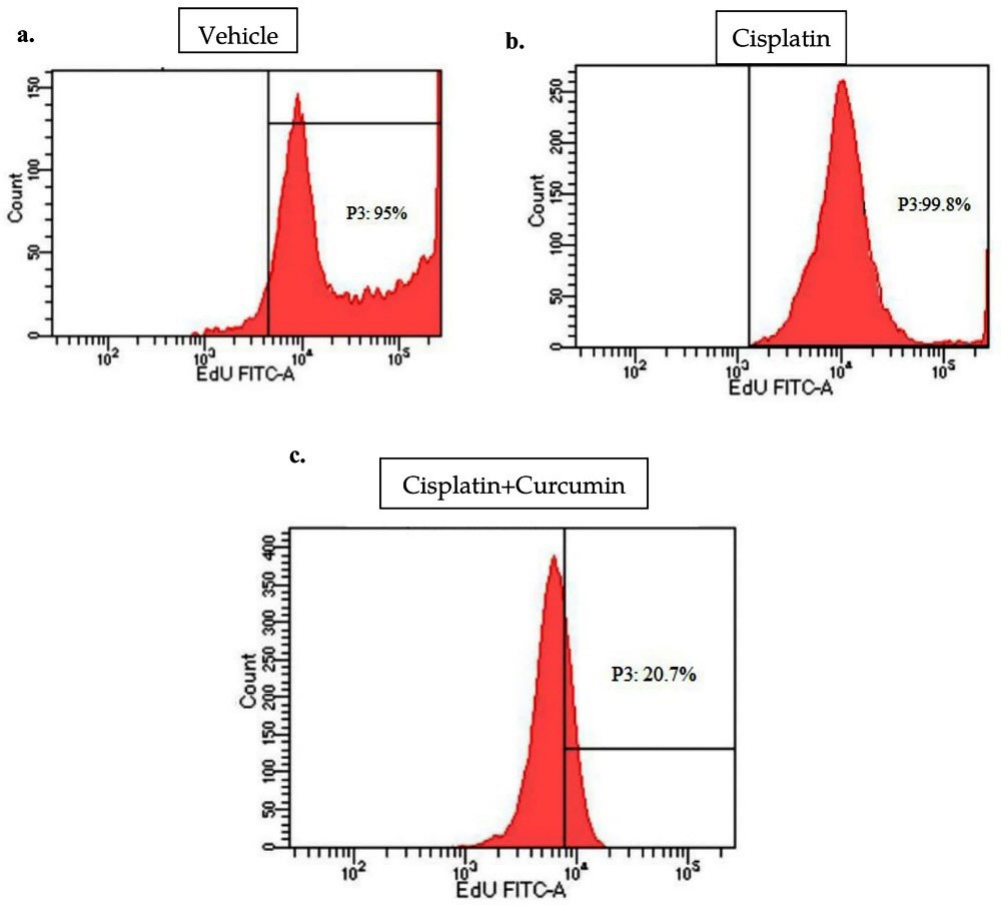
Flow cytometry analysis for cell viability of SKOV3 cell treated with cisplatin ± curcumin by EdU assay. Under treatment of vehicle (a), cisplatin (b), and cisplatin with curcumin (c) (n=3, each group).

CUR treatment could be act as an apoptotic inducer in cancer cells.^38^ We, therefore, investigated whether CUR inhibits tumour growth in SKOV3 cell using EdU Flow Cytometry assay and found that co-treatment of CUR with CIS reduced the viability of SKOV3 ovarian cancer cells compared with those in CIS group alone or vehicle (VEH) group (Figure 2a-c).

**Figure 3:**
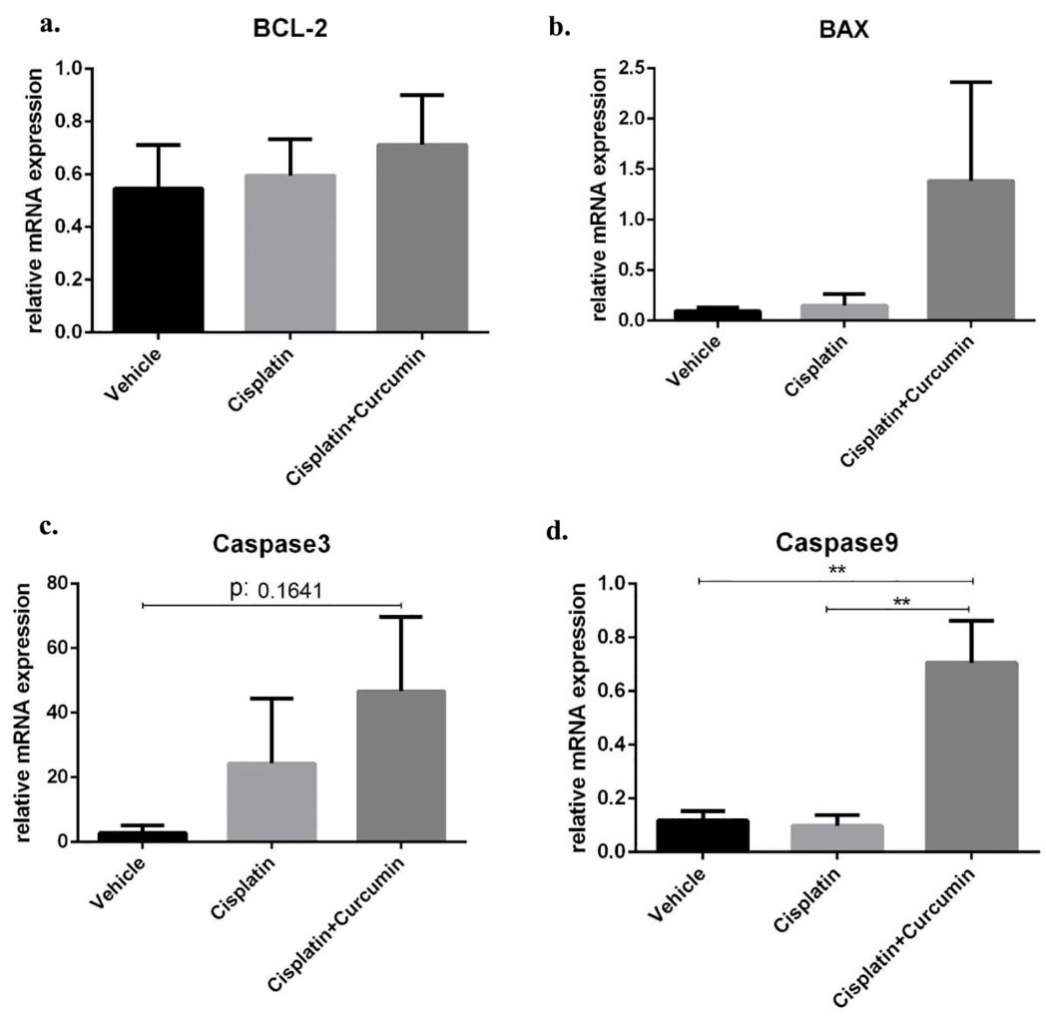
Apoptotic marker at mRNA level in SKOV3 cells after cisplatin ± curcumin treatment. (a) Bax expression marker, (b) BCL-2 expression marker, (c) caspase3 expression marker, (d) caspase9 expression marker. Non-repeated ANOVA with post hoc analysis of Fisher’s PLSD was used for statistical analysis. Data are presented as mean ± SE. *P < 0.05, **P < 0.01, *** P < 0.001, and **** P < 0.0001 (n=4, each group).

Although there was no different of BCL-2 mRNA expression level after treatment of CIS or CUR (Figure 3a). However, CUR supplementation together with CIS (CIS+CUR) enhanced BAX, caspase 3, and caspase9 mRNA expression levels (Figure 3b-d) as apoptosis marker gene at the molecular level indicated that CUR has anticancer properties by increasing apoptosis in SKOV3 ovarian cancer cells. Consistent with our result, CUR altered growth capacity in urothelial and colorectal cancer cell line and regulated BAX/BCL-2 and caspase signalling in various cancer models.^38^

### EMT phenotype induced by heat stress was alleviated by CUR treatment

**Figure 4:**
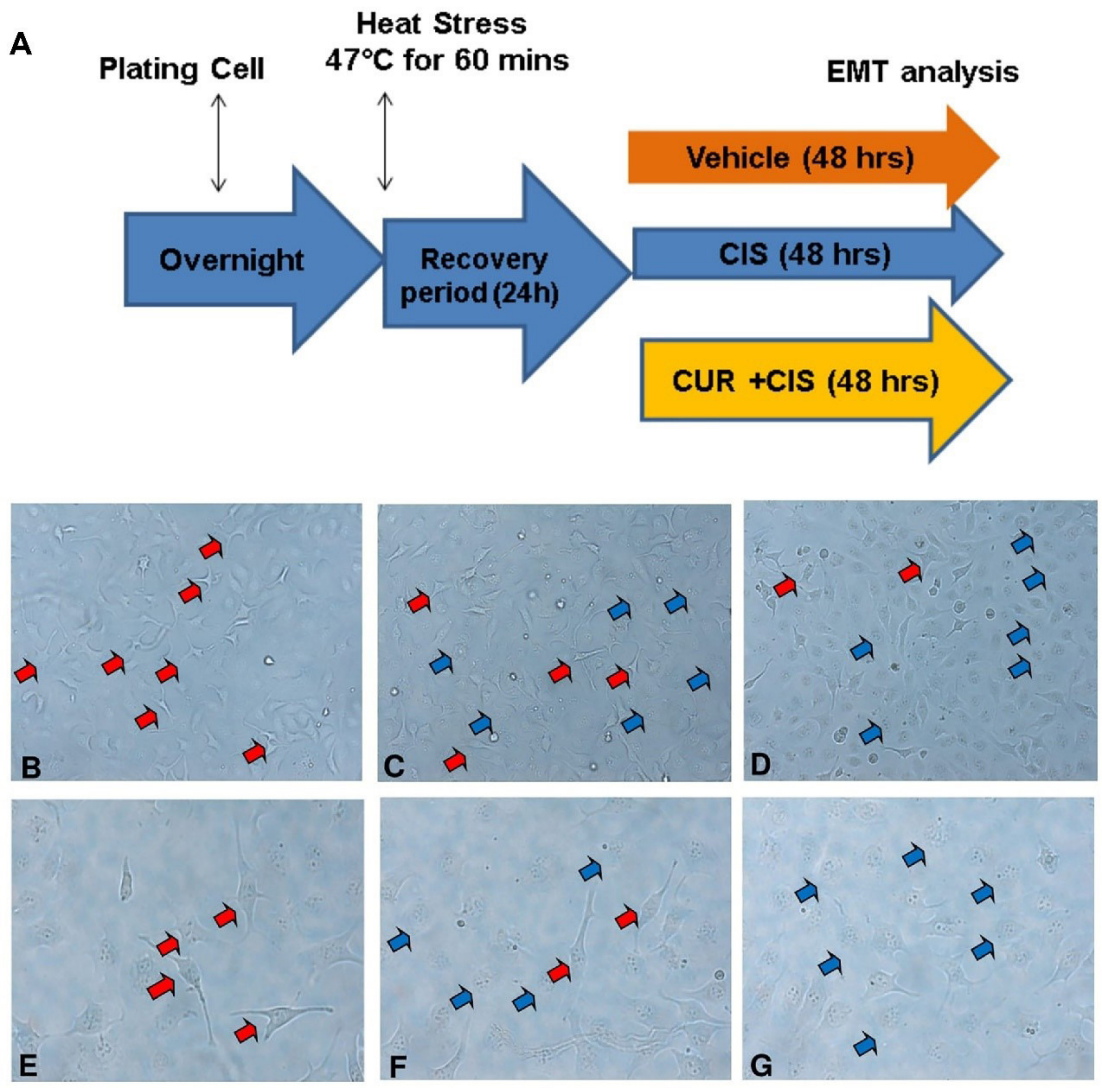
The SKOV3 cell morphology in cisplatin ± curcumin administration after heat shock treatment. (A) Timeline study of heat shock treatment in SKOV3 cell, with VEH at 10x mag (B) and 20x mag (E), with CIS at 10x mag (C) and 20x mag (F), and with CIS+CUR at 10x mag (D) and 20x mag (G). Red arrow indicates spindle shaped cell appearance and blue arrow indicates cobble-stone appearance. VEH: Vehicle treatment; CIS: Cisplatin treatment; CUR: Curcumin treatment.

**Figure 5:**
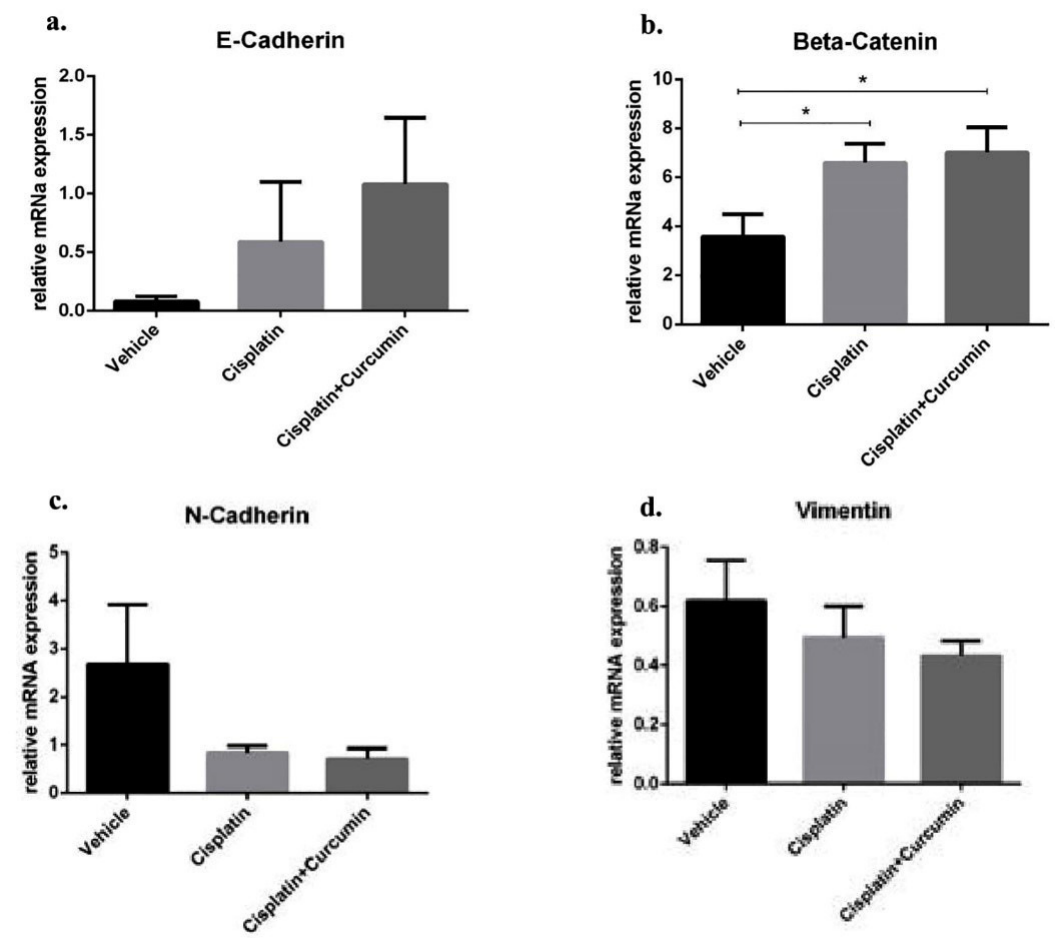
Epithelial markers (E-cadherin and β-catenin) and mesenchymal markers (N-cadherin and vimentin) at mRNA level in SKOV3 cells after cisplatin ± curcumin treatment. (a) E-cadherin expression marker, (b) β-Catenin expression marker, (c) N-cadherin expression marker, (d) Vimentin expression marker. Non-repeated ANOVA with post hoc analysis of Fisher’s PLSD was used for statistical analysis. Data are presented as mean ± SE. *P < 0.05, ** P < 0.01, *** P < 0.001, and **** P < 0.0001 (n=4, each group).

EMT has been responsible for the expansion and growth of cancer, including ovarian malignancy and further increase the mortality chance of malignancy.^6,39^ On the other hand, the inadequate thermal treatment produced by radio-frequent therapy can surprisingly promote EMT process.^40^ In line with that theory, several cell culture studies showed the heat stress at range 42-47°C for 30-60 minutes followed with a recovery period could induce mesenchymal transformation in a cancer cell and further increase tumour invasiveness.^34,41,42^ Moreover, we performed heat stress at SKOV3 cells by incubating at 47°C for 60 minutes and recover it at normal culture condition for recovery.^34^ Afterwards, the cells treated with CIS ± CUR until 48 hours (Figure 4A). When we induced the heat stress procedure as mentioned above, we found the cells increased formation of pseudopodia the spindle shape appearance with reduced of cell-cell contact at VEH group (Figure 4B, E) and CIS treatment (Figure 4C, F) was not sufficient to prevent the mesenchymal transition. However, adjuvant CUR therapy was likely to suppress the EMT phenotype in SKOV3 cell. (Figure 4D, G) Consistently, mRNA analysis showed the increased of mesenchyme markers such as N-cadherin and vimentin with the reduction β-catenin and E-cadherin as epithelial markers in VEH group. Even CIS and CIS+CUR groups were not enhanced E-cadherin expression level dramatically, but those groups significantly enhanced β-catenin expression level. (Figure 5a, b) On the other hand, both CIS and CIS+CUR groups reduced N-Cadherin even not statistically different, and there was no different of vimentin expression level among groups. (Figure 5c, d).

### CUR suppressed ET-1 expression level through ETBR regulation in SKOV3 cells

ET-1 has recognized as the inducer for EMT phenotype-mediated tumour progression in ovarium which displayed by loosens cell and inability to keep the epithelial phenotype.^10,43,44^ Therefore, macitentan and zibotentan (ZD4054), the ETAR antagonist drugs, which are not only to impede the transformation of the epithelial cells and prevent the EMT-mediated metastasis but also to increase the sensitivity of anti-tumour medications, such as CIS and paclitaxel.^45,46^

**Figure 6:**
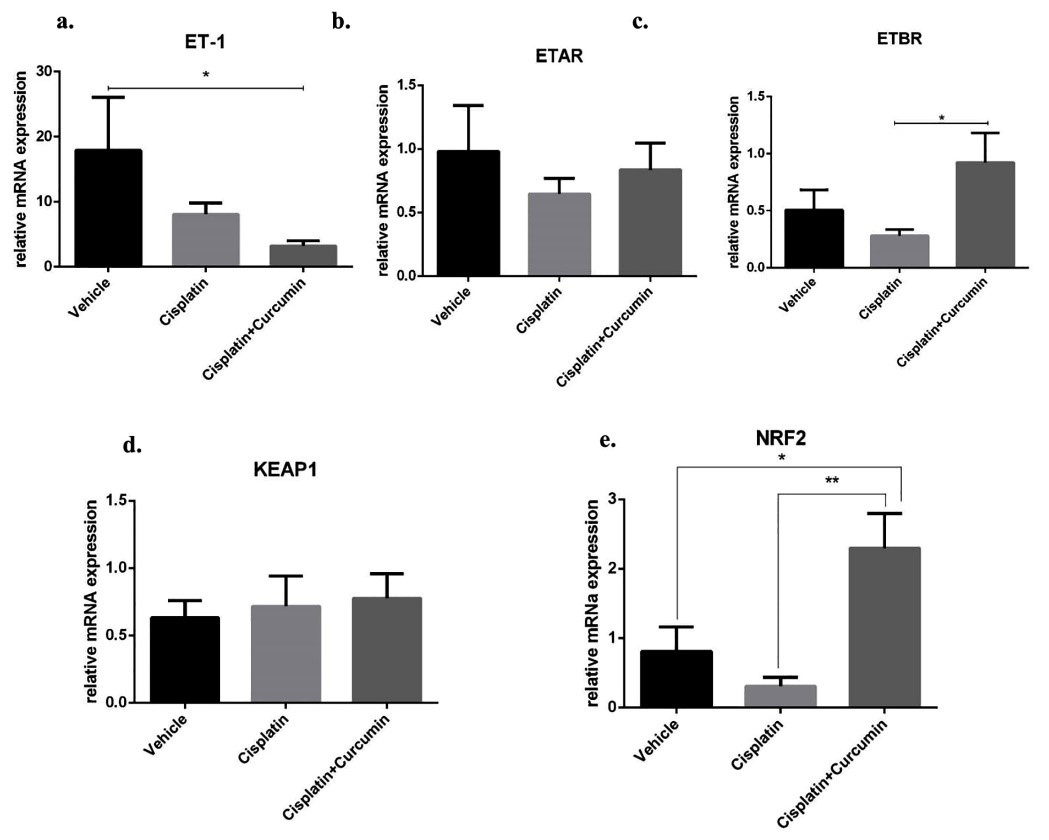
Endothelin-1 (ET-1) and its receptors gene expression and KEAP1/NRF2 gene expression at mRNA level in SKOV3 cells after cisplatin ± curcumin treatment. (a) Endothelin-1 expression marker, (b) ETAR expression marker, (c) ETBR expression marker, (d) KEAP1 expression marker, (e) NRF2 expression marker. Non-repeated ANOVA with post hoc analysis of Fisher’s PLSD was used for statistical analysis. Data are presented as mean ± SE. *P < 0.05, **P < 0.01, *** P < 0.001, and **** P < 0.0001 (n=4, each group).

After thermal treatment, we found that SKOV3 cell enhanced ET-1 expression level and CUR substantially ameliorated those expression level (Figure 6a). Moreover, either CIS or CIS+CUR failed to reduce ETAR expression level (Figure 6b). Despite the direct response of CUR in ETAR expression level, CUR interestingly enhanced ETBR expression level at mRNA level (Figure 6c). Together with the previous result, these data suggest that CUR ameliorated ET-1-mediated EMT phenotype due to ETBR activation in ovarian cancer.

### CUR enhanced NRF2 expression level in SKOV3 cell

Moreover, we next sought the underlying molecular mechanism of ETBR activation after induction of CUR in SKOV3 cell. Nuclear factor erythroid-2-related factor 2 (NRF2) has been known able to directly bind ETBR and further increase ETBR expression level and leading into ET-1 clearance in endothelial cell.^14^ Therefore, we hypothesized that KEAP1-NRF2 signalling could induce the transcription of ETBR in ovarian cancer. Although CUR did not reduce Kelch-like ECH-associated Protein1 (KEAP1) as a negative regulator for NRF2 at mRNA level (Figure 6d), CUR adjuvant enhanced NRF2 expression level in SKOV3 cell, and these phenomena did not occur in CIS treated alone (Figure 6e). These data suggest CUR enhance NRF2-mediated ETBR transcription and reduced ET-1 expression level, thus inhibited tumour growth in SKOV3 cell. Similarly, we and other groups have shown that CUR activated NRF2 expression gene and suppressed ET-1 signalling.^23-25,27,47,48^

### Apoptosis marker analysis in CUR treated-ovarian cancer rat model

**Figure 7:**
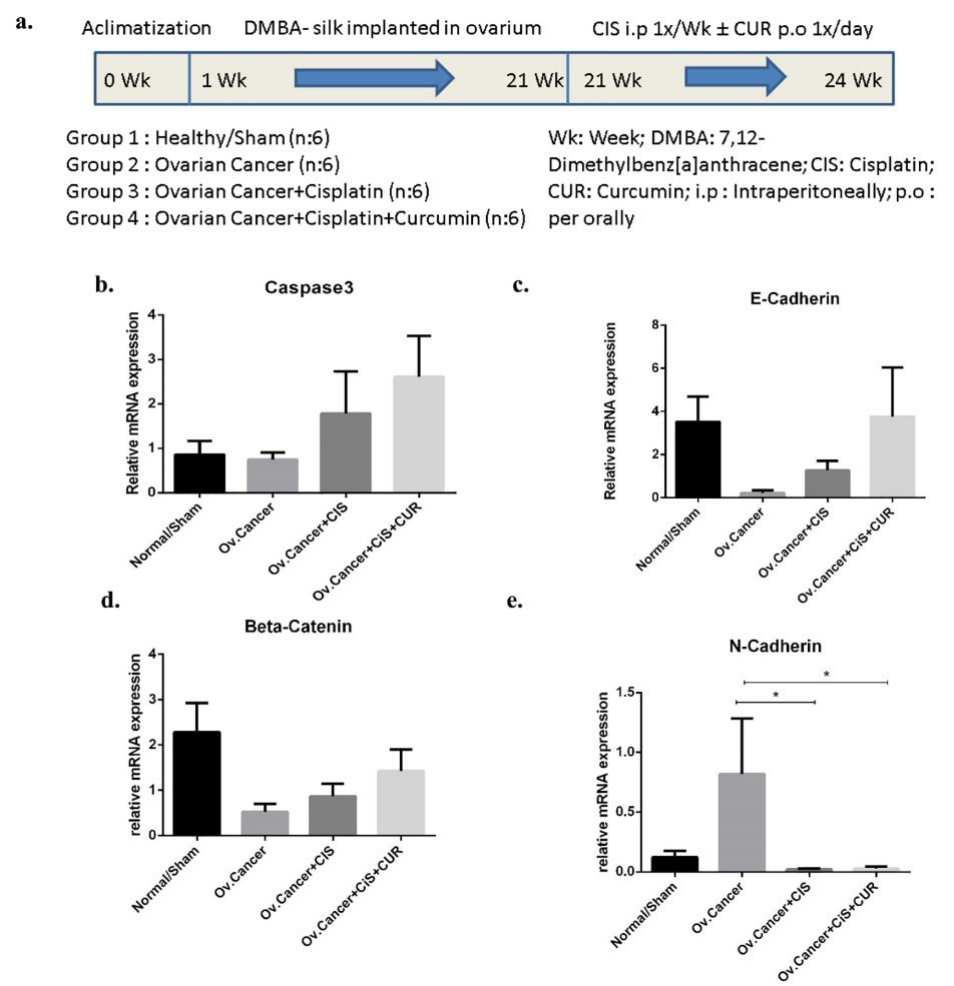
Caspase3 and epithelial-to mesenchymal transition (EMT) markers gene expression at mRNA level in ovarium derived from ovarian cancer rat model treated with cisplatin ± curcumin. (a) The Timeline of in vivo study, (b) Caspase3 expression marker, (c) E-Cadherin expression marker, (d) β-Catenin expression marker, (e) N-Cadherin expression marker. Non-repeated ANOVA with post hoc analysis of Fisher’s PLSD was used for statistical analysis. Data are presented as mean ± SE. *P < 0.05, ** P < 0.01, *** P < 0.001, and **** P < 0.0001 (n=6, each group).

To further confirm the role of CUR on the progressivity of tumour in vivo, ovarian cancer model in the rat was performed as we previously established^35^ by directly implanting silk coated with 7,12-dimethylbenz[a]anthracene (DMBA) into one side of ovarium for each rat until 20 weeks and followed with CIS ±CUR treatment for four weeks. (Figure 7a). We found that caspase3 mRNA expression level was increased in ovarium from DMBA-implanted rat with CIS and CUR compared with those in other groups even not statistically different (Figure 7b).

### Mesenchymal formation was inhibited with CUR in ovarian cancer rat model

Consistent with the in vitro result, we showed the epithelial markers at mRNA levels such as E-cadherin and beta-catenin were reduced together with enhanced of N-cadherin level as mesenchymal marker in ovarium from DMBA+vehicle-treated rat group (VEH). However, E-cadherin and β-catenin mRNA expression levels were improved in ovarium isolated from CIS+CUR groups but not happened in CIS rat group (Figure 7c, d). However, N-cadherin expression level significantly ameliorated in either CIS or CIS+CUR groups (Figure 7e).

### CUR suppressed endothelin signalling in ovarian cancer rat model

**Figure 8:**
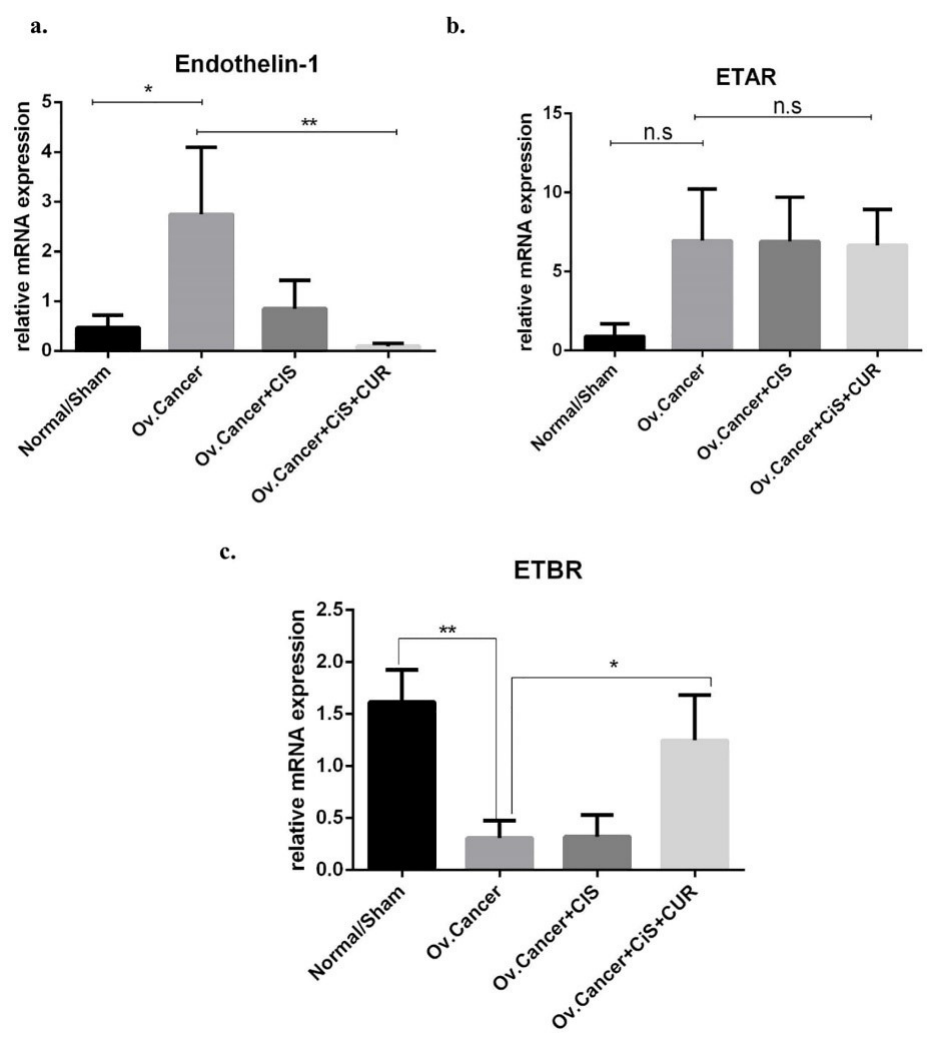
Endothelin-1 (ET-1) and its receptor markers gene expression at mRNA level in ovarium derived from ovarian cancer rat model treated with cisplatin ± curcumin. (a) Endothelin-1 (ET-1) expression marker, (b) ETAR expression marker, (c) ETBR expression marker. Non-repeated ANOVA with post hoc analysis of Fisher’s PLSD was used for statistical analysis. Data are presented as mean ± SE. *P < 0.05, ** P < 0.01, *** P < 0.001, and **** P < 0.0001 (n=6, each group).

**Figure 9:**
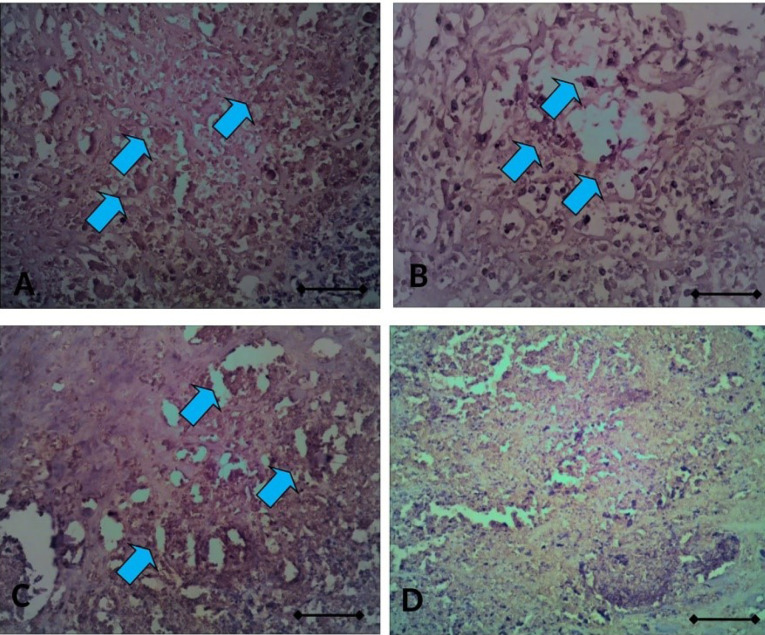
The sections of ovarium rat with ETAR immuno-staining, taken from ovarium rats coated with DMBA-silk; (A) with Vehicle, (B) with CIS, (C) with CIS+CUR, and (D) normal rat. The light blue arrow indicates the immune-positive cell. Scale bar denotes 200x magnification.

The elevation of ET-1 level was detected in ovarian cancer, and ET-1 axis currently has been investigated as a central player for driving EMT in ovarian cancer cells and induced invasive phenotype in ovarian cancer.^49^ ET-1 mediated reduction of the epithelial markers including β-catenin and E-cadherin with mesenchymal phenotypes exacerbation such as N-cadherin and vimentin, thus enhanced cell invasiveness.^10,43,49^ Therefore, ET-1, ETAR, and ETBR expression at mRNA levels were analyzed on the ovarian tumour from rat-treated with VEH, CIS, or CIS+CUR. ET-1 mRNA expression level significantly enhanced in the ovarium from VEH group, and CIS+CUR group displayed a dramatically reduced of ET-1 expression level compared with those in other groups (Figure 8a). Similar to the *in vitro* result, ETAR mRNA expression level was at a comparable level among the groups (Figure 8b), and ETBR mRNA expression level substantially enhanced in the CIS+CUR group. (Figure 8b). We further confirmed that CUR was not altered ETAR expression at Immuno-histochemical staining analysis (Figure 9). These data revealed that CUR adjuvant suppressed ET-1 by interestingly activating ETBR in ovarian cancer, suggesting CUR prevents EMT phenotype by regulating ET-1 signalling.

### CUR improved NRF2 expression level in ovarian cancer rat model

**Figure 10:**
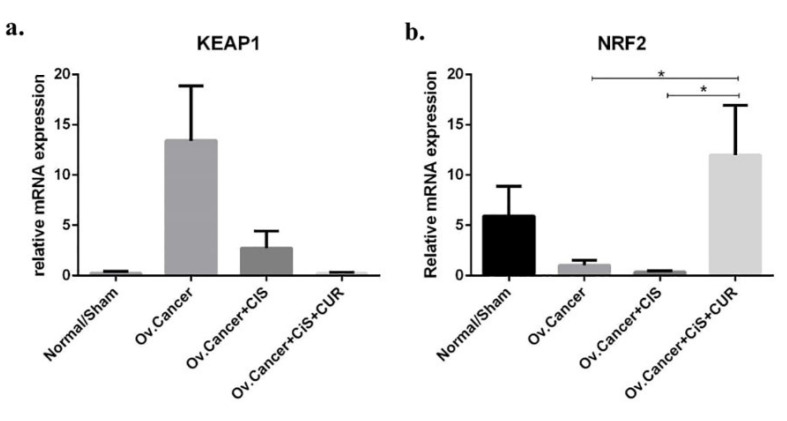
KEAP1/NRF2 gene expression at mRNA level in ovarium dervied from ovarian cancer rat model treated with cisplatin ± curcumin. (a) KEAP1 expression marker, (b) NRF2 expression marker. Non-repeated ANOVA with post hoc analysis of Fisher’s PLSD was used for statistical analysis. Data are presented as mean ± s.e. *P < 0.05, ** P < 0.01, *** P < 0.001, and **** P < 0.0001. (n=6, each group).

Most importantly, we investigated whether the consistent result of NRF2 from cell culture experiment could also be obtained *in vivo*. A reduction of KEAP1 mRNA expression level was detected in CIS+CUR group, whereas CUR sufficiently improved NRF2 expression level in CIS+CUR group. (Figure 10a, b). We, therefore, concluded together with the *in vitro* result that CUR improved NRF2 expression level and activated ETBR gene-mediated ET-1 clearance leading to inhibition of tumour development.

### Kidney structure and function in repeated low CIS treatment in ovarian cancer rat model

**Figure 11:**
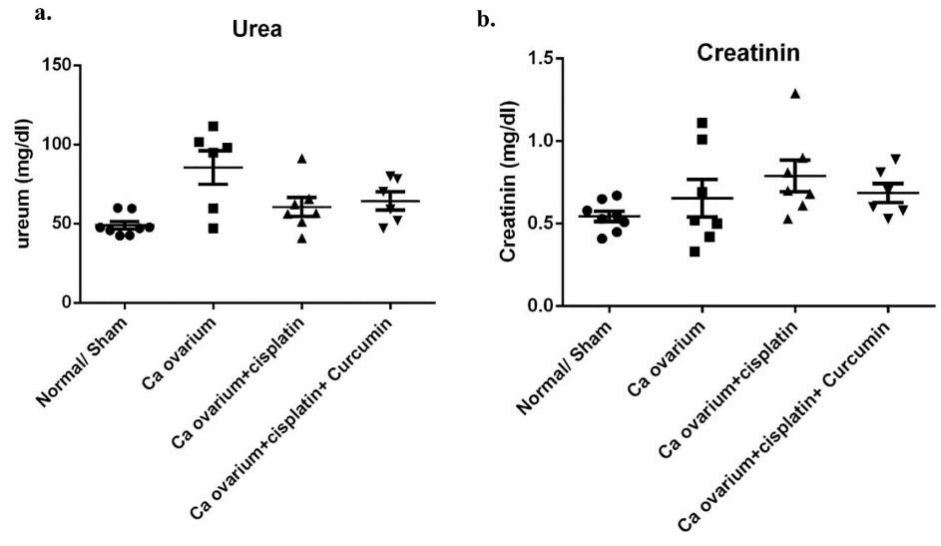
Urea and creatinin plasma level in ovarian cancer rat model treated with cisplatin ± curcumin. (a) Urea plasma level, (b) Creatinin plasma level. Data are presented as mean ± SE. *P < 0.05, ** P < 0.01, *** P < 0.001 and **** P < 0.0001 (n=6, each group).

**Figure 12:**
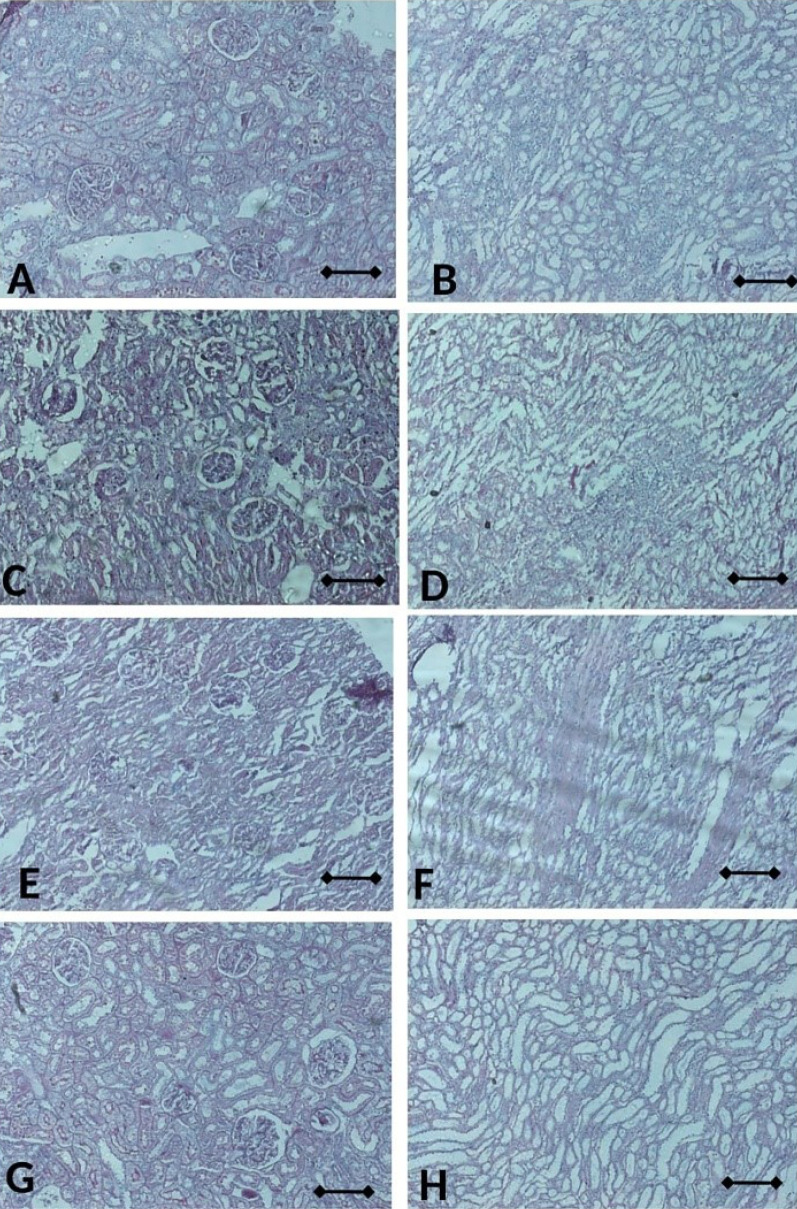
The sections of kidney rat stained with PAS; with Vehicle in glomerulus (A) and tubulus (B), with CIS in glomerulus (C) and tubulus (D), with CIS+CUR in glomerulus (E) and tubulus (F), and normal rat in glomerulus (G) and tubulus (H). Scale bar denotes to 200x mag. CIS: Cisplatin, CUR: Curcumin.

**Figure 13:**
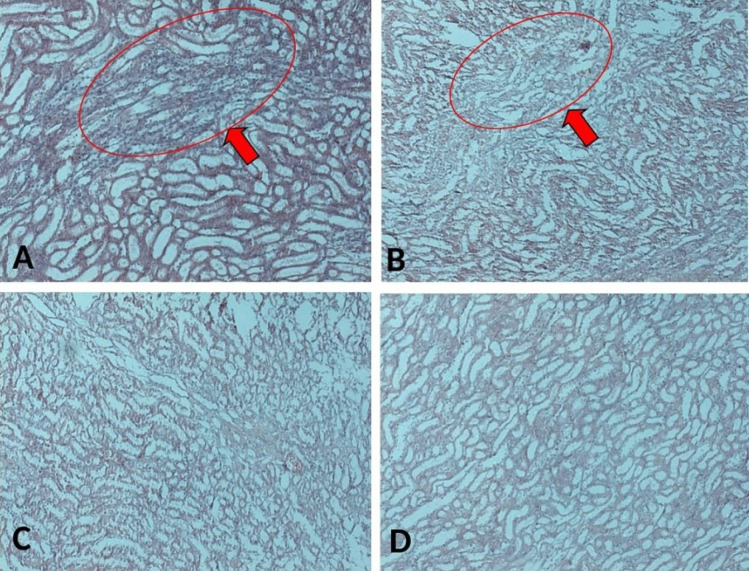
The sections of kidney rat stained with Masson Trichrome, taken from kidney rats induced with ovarian cancer; (A) with Vehicle, (B) with CIS, (C) with CIS+CUR, and (D) normal rat. Red arrow points to the fibrosis. Scale bar denotes 200x magnification.

**Figure 14:**
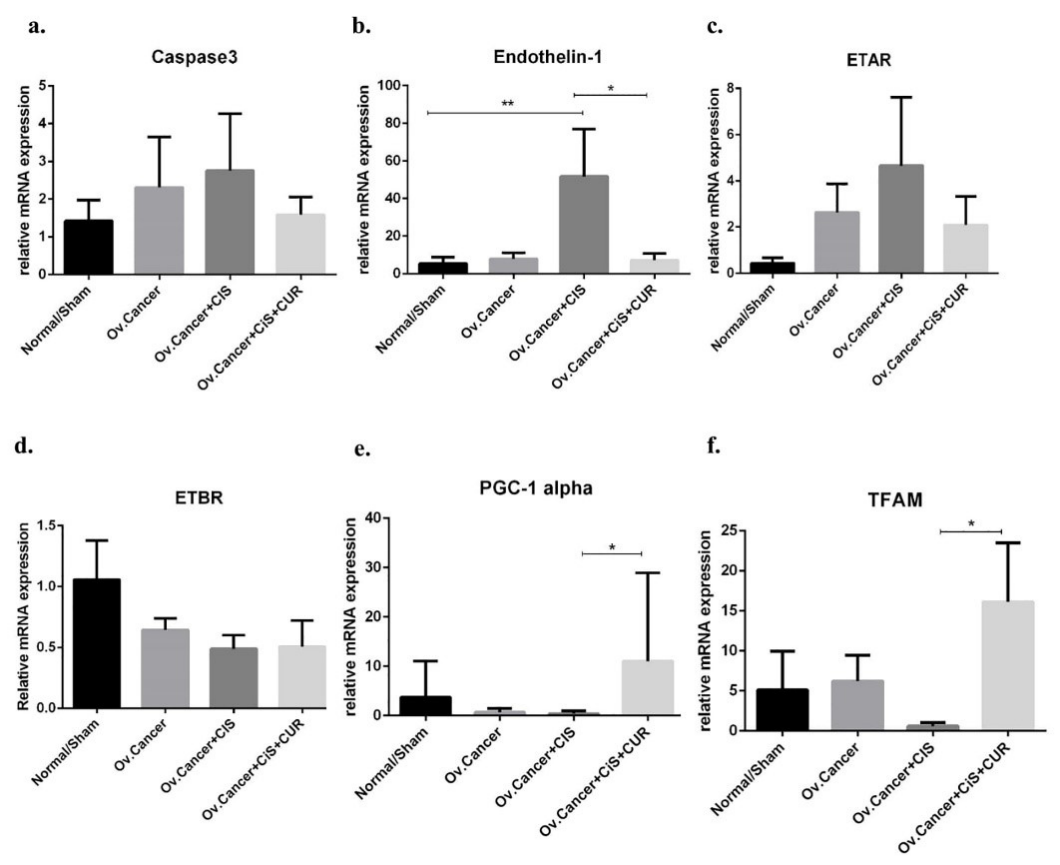
Caspase 3, ET-1/ETAR/ETBR, and mitochondria biogenesis markers gene expression at mRNA level in kidney isolated from ovarian cancer rat model treated with cisplatin ± curcumin. (a) Caspase3 expression marker, (b) ET-1 expression marker, (c) ETAR expression marker, (d) ETBR expression marker, (e) PGC-1α expression marker, (f) TFAM expression marker. Non-repeated ANOVA with post hoc analysis of Fisher’s PLSD was used for statistical analysis. Data are presented as mean ± SE. *P< 0.05, ** P < 0.01, *** P < 0.001, and **** P < 0.0001 (n=6, each group).

Despite a beneficial treatment of CIS in cancer, the superiority of treatment has limited by its toxicity effects on kidney such as elevated of the increased of urea and creatinine levels or localized in the kidney including renal fibrosis and the increased of renal cell apoptosis.^50^ A recent study also shows that CIS can induce renal fibrosis and also both ET-1 and oxidative stress activation at the molecular level even at a therapeutic dose.^51-53^ We next analyzed whether a renal injury was raised in multiple low CIS treatment in our ovarian cancer rat model, and CUR plays as a renoprotective treatment in this model. We found there was a slightly elevated of urea and creatinine plasma levels in DMBA-Cancer group even without CIS treatment (VEH) (Figure 11a, b). This data is consistent with kidney function analysis in the ovarian cancer patient.^54,55^ Moreover, CIS groups also showed similar phenotype with VEH group even not statistically different. Nevertheless, CUR administration has tended to be improved those levels even not statistically different (Figure 11a, b). Consistently, glomerular and tubular structure of kidney by periodic acid-Schiff and Masson’s trichrome staining revealed no significant pathological finding even slight fibrosis detected in both of VEH and CIS group. (Figures 12 and 13). Of note, caspase3 mRNA expression level in the kidney also detected at a comparable level among the groups. (Figure 14a).

### CUR suppressed ET-1 mRNA expression level and preserved mitochondria biogenesis marker in CIS-treated ovarian cancer rat

We presumed that ET-1 axis also plays as a primary regulator for the development of CIS-induced renal injury and CUR could potentially prevent those phenomena through ET-1 suppression. We found that CUR prevented the increased of ET-1 and ETAR mRNA expression level, but ETBR mRNA expression levels were remained similar at CIS+CUR compared with CIS and VEH groups. (Figure 14b-d) In this study, we further specifically revealed the dual function of CUR on ovarian cancer and CIS-induced renal injury by regulating NRF2/antioxidant mechanism-mediated ET-1 axis regulation.

To further elucidate the underlying mechanism of ET-1 activation in CIS-induced renal injury, we analyzed endogenous antioxidant enzyme which responsible for mitochondria biogenesis including Peroxisome proliferator-activated receptor gamma coactivator 1-alpha (PGC-1α) and transcription factor A, mitochondrial (TFAM) since chemo-drug such as doxorubicin has known to disrupt those markers.^56^ Henceforward, we showed that CIS reduced the PGC-1α and TFAM expression levels at mRNA level of in the kidney of VEH and CIS group, and interestingly CUR improved those markers even under CIS treatment (Figure 14e, f). In conclusion, these data revealed that CUR enhance mitochondria biogenesis markers and diminished ET-1 expression level leading into the protection of kidney induced by CIS treatment.^32,33^

It has reported that CUR has anticancer property^21,29^ and also renal injury caused by CIS.^33^ More recently, several ongoing clinical trials have made to identify the beneficial effect of CUR in cancer patients.^20^ The dual function of this turmeric compound in the previous work of Kumar et al.^57^ demonstrated the dual function of CUR as anticancer in breast tumour and reno-protective induced by CIS. However, compared with the previous study, our present finding has several significant findings that essential to be considered. Firstly, we used the modified ovarian cancer model in rodent which we previously found by implanting directly suture silk coated with DMBA into ovarium for 20 weeks and afterwards treated with CIS ± CUR for additionally four weeks. The direct DMBA implantation in ovarium will induce ovarian cancer formation, which mimicking ovarian cancer character in human.^35^ Second, as our understanding, this is the initial study identifying the role of CUR on the regulation of ET-1/ETBR through KEAP1/NRF2 activation in gynaecological malignancy, particularly in ovarian tumour. We showed that CUR inhibits ET-1 through NRF2-mediated ETBR activation. This mechanism hopefully could provide a novel insight into ETBR regulation in ovarian tumour.

Lastly, most of the toxicity effect studies regarding CIS administration in cancer are using high dose in a non-cancer or healthy sample.^50,58^ On the other hand, in the clinical setting, a relatively low and multiple-dose of CIS are frequently used to treat cancer disease. Of note, repeated administration of low dose CIS sufficiently induced renal injury.^51-53^ In this study, multiple low doses of CIS (4 mg/kg BW) weekly,^58^ which equivalent with CIS chemo-treatment in a cancer patient, was sufficiently diminished mitochondria biogenesis markers including PGC-1α and TFAM thus increased ET-1/ETAR expression level even urea/creatinine serum level and histopathology phenotype not altered significantly. Therefore, CUR improved PGC-1α and TFAM in association with ameliorated ET-1 over-activity in kidney isolated from ovarian cancer rat model co-treated with CIS and CUR.

## Conclusion

Our data revealed CUR as a potential natural product for female cancer treatment, including ovarium with additional renoprotective effect against chemotherapeutic such as CIS. As mentioned above, several ongoing clinical trials of CUR are currently performing for various cancers suggesting CUR as a potential of translational herbal medicine in the cancer patient.

We also revealed an unexplored mechanism of chemo-treatment of CUR by regulating NRF2/ETBR/ET-1 signalling pathway. These results support a rational explanation of ET-1 for being an attracting targeted therapy for ovarian cancer; however, the further analysis needed to assess its therapeutic feasibility in vivo due to the low bioavailability of CUR particularly in the ovarium.^59^ Therefore, efficacy therapy of alternative carrier for CUR such as nanoparticle is needed to be analyzed in the future to overcome the insufficient delivery of CUR in the human body.^60,61^ We previously found that nanoparticle of CUR not only enhanced the concentration in plasma but also have better penetration in several organs including ovarium and kidney thus improved its beneficial effect on those organs.^59^

## Ethical Issues

All studies were approved by the Ethics Committee Team of Faculty of Medicine, Universitas Indonesia, Jakarta, Indonesia. (0531/UN2.F/ETIK/2018).

## Conflict of Interest

The authors declare no conflict of interest.

## Funding

This research was funded by Direktorat Riset dan Pengabdian Masyarakat (DRPM) Universitas Indonesia, NKB-0241/UN2.R3.1/HKP.05.00/2019

## Acknowledgments

We thank the Pharmacokinetic Laboratory, Department of Pharmacology and Therapeutics, Faculty of Medicine, Universitas Indonesia; Metabolic, Cardiovascular, and Aging Cluster and Human Reproduction, Infertility, and Family Planning Cluster from Indonesia Medical Education and Research Institute (IMERI), Faculty of Medicine, Universitas Indonesia for kindly supporting the technical issues in this study. We also thank the Direktorat Riset dan Pengabdian Masyarakat (DRPM), Universitas Indonesia for providing Q1Q2 grant for this study

## Supplementary Materials


Supplementary file 1 contains Tables S1-S2.
