# Supplementary material for: Curcumin Prevents Epithelial-to Mesenchymal Transition-Mediated Ovarian Cancer Progression through NRF2/ETBR/ET-1 Axis and Preserves Mitochondria Biogenesis in Kidney after Cisplatin Administration
Source: Adv Pharm Bull. 2020 Sep 19;12(1):128–41. doi: 10.34172/apb.2022.014 (PMC9012927; doi:10.34172/apb.2022.014)
Supplement: Supplementary file 1 — contains Tables S1-S2. [file apb-12-128-s001.pdf]

Supplementary file 1

Table S1. Human Primer

Gene	Sequence	GenBank
β-Actin	F: TTGCGCTCAGGAGGAGCAAT	NM_001101.5
	R: TTCCAGCCTTCCTTCCTGG	
Prepro Endothelin-1 (ppET-1)	F: TCAGCAACAGCATCAAGACC	NM_001955.5
	R: TCCCCAGACAGCAAGAAGAG	
Endothelin A receptor (ETAR)	F: TGATAGCCAGTCTTGCCCTT	NM_001957.4
	R:CTGTACCTGTCAACACTAAGAGCG	
Endothelin B Receptor (ETBR)	F: GGTACTTGGAGTCTGGACATCTGA	NM_000115.5
	R: AGGTCTTAGTGGGTGGCGTC	
KEAP-1	F: TTCGCCTACACGGCCTC	NM_203500.2
	R: GAAGTTGGCGATGCCGATG	
NRF2	F: TCAGCGACGGAAAGAGTATGA	NM_006164.5
	R: CCACTGGTTTCTGACTGGATGT	
Caspase3	F:GGTTAACCCGGGTAAGAATGTGCA	NM_004346.4
	R: TCGGTCTGGTACAGATGTCGAT	
Caspase9	F: CGGAAGCGGACTGAGGCGGC	NM_001278054.1
	R: CCAATGTCCACTGGTCTGG	
Bax	F: CATGTTTTCTGACGGCAACTTC	NM_001291428.2
	R: AGGGCCTTGAGCACCAGTTT	
BCL-2	F: TCACTTGTGGCCCAGATAGG	NM_000633.2
	R: AGATGTCCAGCCAGCTGCAC	
E-Cadherin	F: CGGGAATGCAGTTGAGGATC	NM_004360.5
	R: AGGATGGTGTAAGCGATGGC	
Vimentin	F: AGGAGGAGATGCTTCAGAGAGA	NM_003380.5
	R:CCACTTTGCGTTCAAGGTCAAGAC	
N-Cadherin	F: GGCATCAGGCTCCACAGT	NM_001792.5
	R: GGTGGAGGAGAAGAAGACCAG	
β-Catenin	F: GAGCCTGCCATCTGTGCTCT	NM_001330729.2
	R: AC CAAAGGTGCATGATTTG	

Table S2. Rat Primer

Gene	Sequence	GenBank
β-Actin	F: TGTTGTCCCTGTATGCCTCT	NM_031144.3
	R: TAATGTCACGCACGATTTCC	
Prepro Endothelin-1 (ppET-1)	F: GTTGCTCCTGCTCCTCCTTG	NM_012548.2
	R: GCATGGAGAGCGCAGAGTTG	
Endothelin A receptor (ETAR)	F: TTCCCTCTTCACTTAAGCCGAA	NM_012550.2
	R: GCAACAGAGGCATGACTGAAAA	
Endothelin B Receptor (ETBR)	F: TTGCTCGCAGAGGACTGGCCA	NM_017333.1
	R: AAGCATGCAGACCCTTAGGGG	
KEAP-1	F: CAGCGTGCTCGGGAGTAT	NM_057152.2
	R: GTGTGACAGGTTGAAGAACTCCT	
NRF2	F: AGCATGATGGACTTGGAATTG	NM_031789.2
	R: CCTCCAAAGGATGTCAATCAA	
Caspase3	F: AAATTCAAGGGACGGGTCAT	NM_012922.2
	R: ATTGACACAATACACGGGATCTGT	
PGC-1α	F: ATGTGTCGCCTTCTTGCTCT	NM_031347.1
	R: ATCTACTGCCTGGGGACCTT	
TFAM	F: GCTAAACACCCAGATGCAAAA	NM_031326.1
	R: CGAGGTCTTTTTGGTTTTCC	

Word Abbreviation List

EMT : Epithelial-to Mesenchymal Transition, ET-1: Endothelin-1, ETAR: Endothelin A Receptor, ETBR: Endothelin B Receptor, GPCRs: G-Protein Coupled Receptors, ERA: Endothelin Receptor Antagonist, CIS: Cisplatin, CUR: Curcumin, VSMC: Vascular Smooth Muscle Cell, EC: Endothelial Cell, KEAP1: Kelch-like ECH-Associated Protein 1, NRF2: Nuclear factor erythroid-2-related factor 2, PGC-1α: Peroxisome Proliferator-Activated Receptor-Coactivator 1 α (PGC-1α), TFAM: Mitochondrial Transcription Factor a
